# Retained Stingray Barb in the Sole of the Foot

**DOI:** 10.5811/cpcem.63973

**Published:** 2026-08-06

**Authors:** So Sakamoto

**Affiliations:** Asahi General Hospital, Department of Emergency and Critical Care Medicine, Chiba, Japan

**Keywords:** stingray injury, retained foreign body, foot puncture wound, marine envenomation, radiography

## Abstract

**Case Presentation:**

A 46-year-old woman presented to the emergency department after stepping on an object while playing in shallow seawater. Examination revealed a barbed foreign body protruding from the plantar aspect of the left foot. Plain radiography demonstrated that the retained stingray barb had fragmented into three pieces within the soft tissue. Because deeper extension was a concern, computed tomography was additionally obtained to better assess the depth and extent of penetration. The fragments were removed through staged incisions, and repeat radiography confirmed complete extraction. The wound was irrigated, a Penrose drain was placed, and levofloxacin was prescribed at discharge.

**Discussion:**

Stingray injuries are among the most common marine vertebrate envenomations and should not be regarded as simple puncture wounds when retained foreign body is suspected. This case highlights that even when a stingray barb is externally obvious, internal fragmentation may alter procedural planning. Radiography can reveal retained fragments, and additional imaging may be useful when injury depth is uncertain.

## CASE PRESENTATION

**Image 1:**
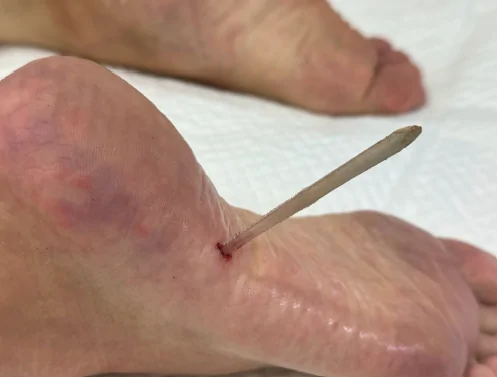
Barbed foreign body protruding from the plantar aspect of the left foot after marine injury.

A 46-year-old woman presented to the emergency department (ED) with pain and swelling of the left foot after stepping on an object while playing in shallow seawater. Physical examination revealed a barbed foreign body protruding from the plantar aspect of the foot (Image 1). The surrounding soft tissue was swollen and tender. Her vital signs were as follows: blood pressure, 113/mm Hg; pulse, 77 beats per minute; respiratory rate, 16 breaths per minute; oxygen saturation, 98% on room air; and body temperature 35.9 °C. She was alert and fully oriented.

**Image 2:**
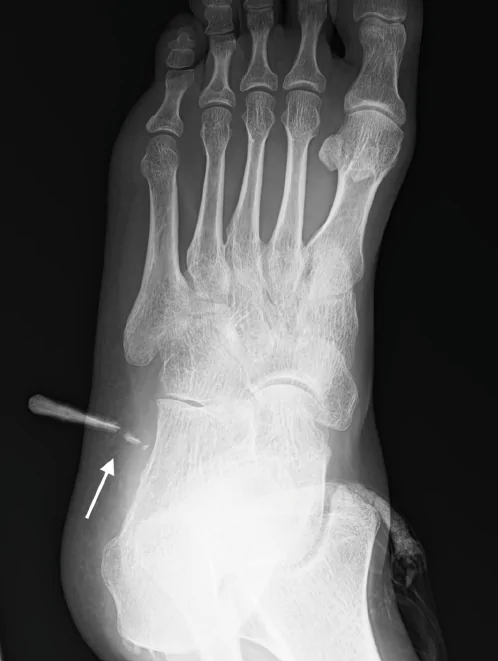
Plain radiograph of the left foot demonstrating a retained stingray barb fragmented into three pieces within the soft tissue.

Plain radiography demonstrated a retained stingray barb penetrating the left foot with fragmentation into three pieces within the soft tissue (Image 2). Because the injury involved the plantar foot and deeper extension was a concern, computed tomography was additionally obtained to better assess the depth and extent of penetration.^1^,^2^ An incision was first made on the plantar aspect of the foot, and the largest fragment was removed. Because two additional fragments remained dorsolaterally, a separate zigzag incision was made directly over the dorsolateral aspect of the foot, and the remaining two fragments were extracted. Repeat radiography confirmed no residual retained foreign body.

**Image 3:**
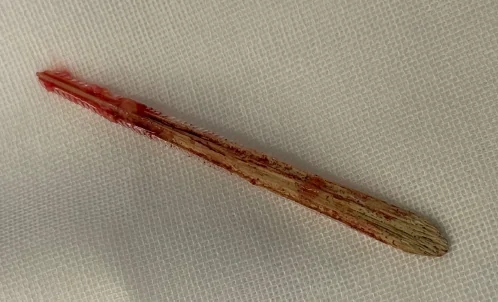
Removed stingray barb showing characteristic backward-facing serrations.

The extracted foreign body was identified as a stingray barb with characteristic backward-facing serrations (Image 3). The wound was then copiously irrigated, a Penrose drain was placed, and the wound was loosely closed. Levofloxacin was prescribed at discharge, and the patient was sent home with follow-up instructions. Because she returned to her home region, additional follow-up information was not available.

## DISCUSSION

Stingray injuries are among the most common marine vertebrate envenomations and most often involve the foot or lower extremity after accidental contact in shallow coastal water.^3^,^4^ These injuries should not be treated as simple puncture wounds because retained barbs may lead to persistent pain, delayed healing, infection, and local tissue injury.^1^,^3^

This case illustrates an important procedural point: Even when a stingray barb is externally visible, internal fragmentation may not be apparent on inspection alone. In our patient, radiography demonstrated that the barb had fragmented into three retained pieces, which changed management and required staged extraction through separate incisions. Computed tomography provided additional assessment of penetration depth, and repeat radiography was useful to confirm complete removal.^1^,^2^

*CPC-EM Capsule*

What do we already know about this clinical entity?

*Stingray injuries are a common marine envenomation and may cause retained barbs, soft tissue injury, infection, and delayed healing.*

What is the major impact of the image(s)?

*The images show an externally visible stingray barb, radiographic evidence of retained fragmentation, and the extracted serrated barb.*

How might this improve emergency medicine practice?

*Even when a stingray barb appears obvious on inspection, imaging may reveal internal fragmentation that changes procedural planning and supports complete removal.*

Superficial retained barbs may sometimes be removed during bedside wound exploration; however, deeper injuries, more complex retained fragment patterns, or concern for involvement of critical structures should prompt more cautious evaluation and consideration of specialist or operative management rather than simple traction removal in the ED.^1^,^3^

Prophylactic antibiotics are not necessary for every stingray injury, but they are reasonable in deeper penetrating wounds, retained foreign bodies, and more contaminated marine wounds.^3^,^5^ Because marine wound infections may involve saltwater organisms in addition to usual skin flora, the exposure environment should be considered when selecting antimicrobial coverage.^3^,^5^ In this case, levofloxacin was prescribed after removal of multiple retained fragments from a deep plantar wound.
